# Conjunctivitis as the only sign and symptom of COVID-19: A case report and review of literature

**DOI:** 10.5339/qmj.2021.31

**Published:** 2021-08-31

**Authors:** Hamzeh Mohammad Alrawashdeh, Khalid Al Zubi, Dina M. Abdulmannan, Omar Al-Habahbeh, Luai Abu-Ismail

**Affiliations:** ^1^Ophthalmology Department, Sharif Eye Centers, Irbid, Jordan E-mail: dr_hmsr@yahoo.com; ^2^Department of Special Surgeries, Faculty of medicine, Mutah University, Al Karak, Jordan; ^3^Ophthalmology Department, Faculty of Medicine, Umm Alqura University, Makkah, Saudi Arabia; ^4^Ophthalmology Department, Ibn Al Haytham Hospital, Amman, Jordan; ^5^Department of Clinical Medical Sciences, Faculty of Medicine, Yarmouk University, Irbid, Jordan

**Keywords:** conjunctivitise, coronavirus, SARS-CoV-2, COVID-19, ophthalmologists, healthcare providers.

## Abstract

Similar to several viruses, coronaviruses can affect the eye and cause conjunctivitis. In addition to ocular involvement, it causes systemic manifestations, mainly respiratory symptoms. However, conjunctivitis as the only sign and symptom of coronavirus disease 2019 (COVID-19) is a rare presentation. We present a case of a 20-year-old male patient who presented with conjunctivitis for 3 days and diagnosed on the same day with COVID-19 without other manifestations. Conjunctivitis affected both eyes and resolved over 2 weeks with artificial tears only and without any ocular complications.

Conjunctivitis can be the only sign and symptom of COVID-19 in some patients. Therefore, healthcare providers, particularly ophthalmologists, should take precautions when dealing with patients presenting with conjunctivitis amid the COVID-19 pandemic.

## Introduction

Coronavirus disease 2019 (COVID-19) is an illness caused by *severe acute respiratory syndrome coronavirus 2* (*SARS-CoV-2*), which is a novel virus from single-stranded RNA coronavirus family. Morphologically, coronaviruses are round, enveloped, and medium-sized particles (80–90 nm) covered with a distinctive fringe of widely spaced and club-shaped surface projections giving it their name. It can infect various species, including chickens, cats, dogs, pigs, bats, and humans. Generally, most of the infected patients complain of fever, dry cough, and dyspnea. Less commonly, some patients may have rhinorrhea, sneezing, sore throat, or other upper respiratory tract symptoms.^1,2^

The *SARS-Cov-2* gains access to the host cells by binding to the angiotensin-converting enzyme II (ACE2) receptor present in various tissues.^3^ These receptors are found in the eye, specifically in the cornea, conjunctiva, ciliary body, iris, and retina. The presence of ACE2 receptors in the cornea and conjunctiva would make the entry of *SARS-CoV-2* through the ocular surface theoretically possible.^4^ Signs and symptoms of conjunctivitis due to *SARS-Cov-2* are similar to those of acute viral conjunctivitis, such as conjunctival congestion, follicular reaction, watery eyes, periorbital rash, and lid edema. Conjunctivitis can precede or occur with systemic illness. Moreover, it can be the sole finding without systemic manifestations.^5,6^ Therefore, some patients infected with *SARS-CoV-2* may present with conjunctivitis before developing other symptoms.^5^

## Case Presentation

A 20-year-old male patient, without significant past medical history, presented with diffuse redness in both eyes, photophobia, foreign body and burning sensation, mild upper eyelid swelling, itching, and excessive tearing for 3 days. Apart from the history of close contact with his friend, who was diagnosed with COVID-19 7 days before the presentation, he denied experiencing fever, cough, sore throat, shortness of breath, tiredness, headaches, flu-like symptoms, skin rash, or gastrointestinal symptoms.

**Figure 1.**
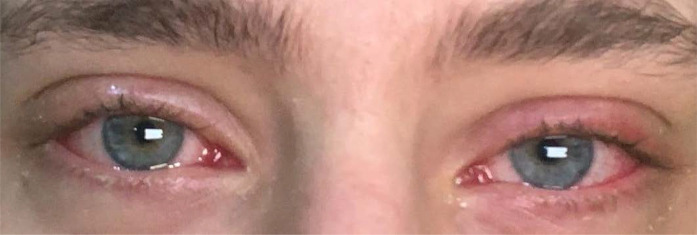
Bilateral conjunctivitis: diffuse conjunctival redness, watery secretions, and mild upper bilateral eyelid swelling.

Upon examination, under coronavirus precautions, his vital signs were within normal limits, there was no preauricular lymph node enlargement, and the best-corrected visual acuity was 20/20 in both eyes. The slit-lamp examination revealed conjunctival injection, thin whitish and watery secretions, and follicular reaction in both eyes (Figure 1). Two swabs were taken from the inferior palpebral conjunctiva in both eyes to identify the presence of *SARS-CoV-2* in the patient's tears. The patient was instructed to get a nasopharyngeal swab to rule out COVID-19 and undergo self-quarantine until the results are available. He was given artificial tears every 2 hours.

The real-time reverse transcription-polymerase chain reaction (RT-PCR) test of the nasopharyngeal and conjunctival swabs was positive for *SARS-CoV-2*. Over the quarantine period, the patient was contacted through the phone. Conjunctivitis improved remarkably, and it was the only manifestation of COVID-19 in this patient. Two weeks from the first presentation, the patient reported complete resolution of conjunctivitis, and the RT-PCR test was negative. He was followed in the clinic at the third and eighth weeks, and the ocular examination was within normal limits in both eyes.

## Discussion

Conjunctivitis is the inflammation or infection of the conjunctiva. It is characterized by dilatation of the conjunctival vessels resulting in conjunctival hyperemia and edema. Typically, conjunctivitis is associated with discharge related to bacterial or viral etiology.^7^ Conjunctivitis due to *SARS-CoV-2* infection presents the same picture as acute conjunctivitis caused by other viruses. Patients may have conjunctival congestion, excessive tearing, lid edema, itching, and photophobia. Conjunctivitis may present with or without systemic manifestations of COVID-19.^5^ Conjunctivitis caused by *SARS-CoV-2* is self-limiting and does not lead to complications such as corneal infiltrates, membranes, and pseudomembranes. It may improve in a few days without specific treatment.^8^

The presence of ACE2 receptors on the ocular surface, which acts as a binding site for SARS-CoV-2, makes the eyes a potential target for *SARS-CoV-2*. Thus, its ability to cause conjunctivitis must be considered.^4,9,10^ The *SARS-CoV-2* RNA has been detected in conjunctival secretions and tears in patients with COVID-19.^10–13^ A study carried out by Güemes-Villahoz showed that the presence of *SARS-CoV-2* RNA in ocular secretions supports the suggestion that the eye is a possible route of infection.^8^ *SARS-CoV-2* is assumed to be transmitted by tears from the conjunctival sac to the respiratory tract through the nasolacrimal ducts. The ability of the virus to bind to the ocular surface and reach the respiratory tract from the eye should prompt healthcare providers to wear eye safety goggles or face shield when they are in close contact with confirmed or suspected cases of COVID-19.^14^ In addition, proper hand hygiene for healthcare providers is of great importance to prevent the transmission of the virus.^15^

Limited studies have reported that conjunctivitis could precede, follow other systemic manifestations, or be the only symptom and sign of COVID-19. A case of acute conjunctivitis followed by respiratory symptoms of COVID-19 was reported in a 65-year-old male patient.^16^ Marquezan et al. reported a case of conjunctivitis preceded by myalgia and fever in a patient with confirmed COVID-19.^17^ Similar to our case, Ozturker reported a case of conjunctivitis as the only symptom of COVID-19 in an emergency healthcare worker.^18^ The RT-PCR test using the nasopharyngeal and conjunctival swabs was positive for *SARS-CoV-2*. Another study reported the case of a 3-year-old child with conjunctivitis and eyelid dermatitis, which were the only manifestations of COVID-19.^19^ Eid and Al Khalaf reported a case of acute conjunctivitis as the only sign and symptom in a patient with COVID-19.^20^ Furthermore, Scalinci and Trovato reported five cases of conjunctivitis as the only sign and symptom of COVID-19.^21^ Moreover, the present case report highlights that conjunctivitis can be the only finding in patients with COVID-19, which is considered a rare presentation.

## Conclusion

Conjunctivitis can be the only manifestation of COVID-19 in some patients. Thus, ophthalmologists and healthcare providers should be aware of this possibility amid the COVID-19 pandemic. They should take extreme precautions and wear eye safety goggles or face shield when dealing with patients presenting with conjunctivitis. Hand hygiene is strongly recommended for patients with conjunctivitis as well as healthcare professionals dealing with those patients. Such measures will decrease the spread of the virus to their airways or to other people.

### Learning Points

• Conjunctivitis can be the only manifestation of COVID-19.

• Extreme precautions should be taken when dealing with patients presenting with conjunctivitis amid the COVID-19 pandemic.

### Ethic Approval And Patient Consent

Written informed consent was obtained from the patient.

### Consent For Publication

Written informed consent was obtained from the patient to publish this case report and accompanying images.

### Conflicts Of Interest

The authors declare no conflict of interest.

### Declarations Of Interest

None.

### Funding

This case report received no external funding.

### Acknowledgements

None.

### Authors’ Contributors

HMR: manuscript construction, revision, literature review, diagnosis, and clinical care. KZ, OH, and LI: manuscript construction, revision, and clinical care. DMA: manuscript construction and revision.
